# Laser Photolysis and Thermolysis of Organic Selenides and Tellurides for Chemical Gas-phase Deposition of Nanostructured Materials

**DOI:** 10.3390/molecules14031111

**Published:** 2009-03-12

**Authors:** Josef Pola, Akihiko Ouchi

**Affiliations:** 1 Laboratory of Laser Chemistry, Institute of Chemical Process Fundamentals, Academy of Sciences of Czech Republic, 16502 Prague, Czech Republic; 2 Research Institute for Innovation in Sustainable Chemistry, National Institute of Advanced Industrial Science and Technology AIST, Tsukuba Central 5, Tsukuba, Ibaraki 305-8565, Japan

**Keywords:** Laser-induced decomposition, Laser-induced deposition, Organoselenium molecules, Organotellurium molecules, Nanostructured Se- and Te- based materials.

## Abstract

Laser radiation-induced decomposition of gaseous organic selenides and tellurides resulting in chemical deposition of nanostructured materials on cold surfaces is reviewed with regard to the mechanism of the gas-phase decomposition and properties of the deposited materials. The laser photolysis and laser thermolysis of the Se and Te precursors leading to chalcogen deposition can also serve as a useful approach to nanostructured chalcogen composites and IVA group (Si, Ge, Sn) element chalcogenides provided that it is carried out simultaneously with laser photolysis or thermolysis of polymer and IVA group element precursor.

## 1. Introduction

Organoselenium and organotellurium chemistry has been developing over the last 50 years owing to studies of the specific roles of the chalcogen atom in organic skeleta and the search for synthetic applications of organoselenium and organotellurium reagents [1,2]. Thermolytic and photolytic fragmentations of the C-X bonds (X=Se, Te) received considerable attention in mechanistic studies and studies of chemical transformations important for synthetic applications [3,4,5,6,7,8]. These fragmentations were mostly carried out in the liquid phase at elevated temperatures or by using conventional (lamp) sources of UV/Vis radiation. Those of compounds with C-X_n_-C bonds (X=Se, Te; n = 1,2) not containing any other heteroatom in carbon skeleton lead to formation of elemental chalcogen. This process is controlled by the C-Se [9] and C-Te [10,11] bond dissociation energies and relative stabilities of Se- and Te-centered radicals [6] and it is more feasible for Te than Se.

Thus, the photolysis or thermolysis of diselenides (involving both C-Se and Se-Se cleavage) and selenides often yields a mixture of hydrocarbons, selenide, polyselenides and elemental selenium [4]. A complete Se extrusion can be only achieved under conditions of flash vacuum (gas-phase) pyrolysis of dibenzyl selenides [12] or diselenacyclophanes [13] and in the photolysis of 2-(methoxycarbonyl)ethyl selenide [14], diindolyl selenide [15] or the photolysis of selenides in the presence of phosphine [3,5].

Thermolysis and photolysis of ditellurides yielding elemental tellurium and telluride [16,17,18] and that of tellurides leading to feasible formation of elemental tellurium has less synthetic application. However, pyrolytic extrusion of Te from tellurides makes possible synthesis of carbocyclic systems [19] and cyclobutenes [20] and coupling of allylic halides [21], and photolytic fragmentation of dimethyl telluride [22] enables gas-phase generation of Te atoms that add to alkenes [23].

The advent of laser chemistry about three decades ago proved many advantages of the use of the highly collimated, intense and monochromatic radiation of lasers for inducing chemical reactions of a variety of organic and organometallic compounds [24,25,26]. Interaction of laser radiation with organic selenides and tellurides leading to extrusion of chalcogen has been studied in various arrangements for chemical vapor deposition of elemental chalkogen and materials composed of elemental chalkogen or metal chalkogenide. 

This review is intended to cover the interactions of laser radiation with gaseous organic chalcogenides with regard to their decomposition steps, final volatile products and solid products depositing on the cold substrate. Two approaches involving the laser beam directed parallel to cold substrate surfaces are included. These are the UV laser-induced photolysis and the IR laser-induced homogeneous thermolysis. Both processes make possible the decomposition of organic chalcogenides in a small volume of the gas phase, far from the surface, which circumvents heterogeneous steps so important in conventional pyrolysis of organic chalcogenides. The laser-induced decomposition of organic chalcogenides can therefore give information on products of the decomposition of these compounds in strictly homogeneous conditions and they prove valuable for chemical vapor deposition of several important solid products.

## 2. The nature of laser-induced processes

Interaction of molecules by UV laser radiation allows their electronic excitation and the decomposition from high singlet or triplet, or vibrationally excited ground states [25]. Interaction of molecules with IR laser radiation takes place via collisionless or collisional multiple-photon excitation transferring the absorbing molecule to high vibrational levels of the ground state [24]. For non-absorbing molecules, the IR laser process can be sensitized by an extremely (thermally) stable absorber (e.g. SF_6_) [27].

**Figure 1:**
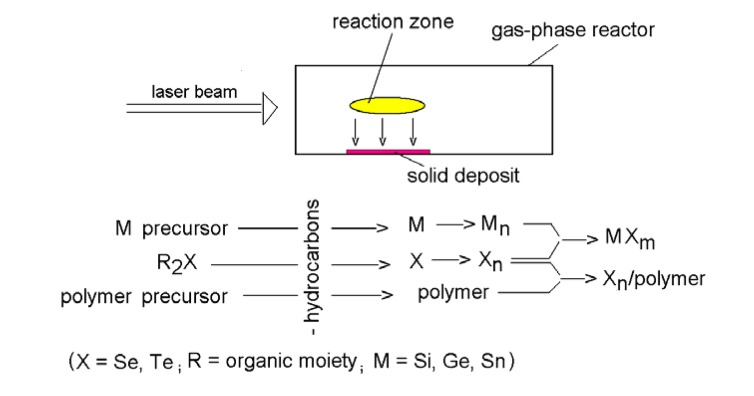
Scheme of decomposition, agglomerization and reaction/interaction between the decomposition products.

The decomposition of organic chalcogenide yields, apart from a number of non-obtrusive volatile hydrocarbons, elemental chalcogen that undergoes agglomerization in the course of its deposition to nanostructured solid powders or thin films. When carried out in the presence of another decomposition, this co-decomposition results in the formation of nanostructured chalcogen-containing materials. Thus. laser photolysis or laser thermolysis of a couple of gaseous precursors (chalcogen precursor with either polymer precursor or metal precursor) allows: (i) simultaneous gas-phase formation of atoms (clusters) of chalcogen and metal, or atoms (clusters) of chalcogen and polymer and (ii) mutual reaction/interaction of these gaseous species, which results in the gas-phase formation and deposition of nanoscopic metal chalcogenide or polymer-encapsulated chalcogen. These possibilities are shown schematically in Figure 1. 

## 3. Deposition of chalcogen

Thermal and photolytic chemical vapor deposition of elemental selenium and tellurium is important due to applications of selenium films in microelectronics and photographic imaging [28,29,30] and the use of low band-gap and infrared transparent tellurium films as shields in passive radiative cooling, gas sensors and optical information storage [31,32,33].

Conventional thermolysis and photolysis of organic selenides and tellurides leading to deposition of selenium and tellurium on hot substrates is accompanied with heterogeneous steps occurring on the reactor surface or substrate. Thus, flash thermolysis of dimethyl selenide yields selenoformaldehyde and methane [34], whereas conventional pyrolysis takes place via homolysis of the C-Se bond, yields several hydrocarbons and is affected by the catalysis of the deposited Se [35]. Surface involvement was also observed in thermal decomposition of dimethyl telluride in a hot tube, which is accelerated by the deposited Te [36]. It is progressively more important in thermolysis of higher dialkyl tellurides where heterogeneous catalysis contributes to simple bond cleavage and complex elimination steps [37,38]. Decomposition of organic tellurides was judged [39] as proceeding via competing C-Te bond homolysis and a β-H-elimination mechanism, yielding RTe**^.^** and RTeH species that rapidly decompose to a hydrocarbon and Te. The flash UV photolysis of dimethyl telluride and diethyl telluride is a usable source of Te atoms that have been spectroscopically detected [22]. 

However, none of these studies involved detailed analysis of hydrocarbon and final solid deposited products; these aspects have been only treated in thermolytic and photolytic studies of organic selenides and tellurides carried out by using laser radiation.

### 3.1. UV laser photolysis

#### 3.1.1. Dimethyl selenide and dimethyl telluride

Visible laser multiphoton dissociation of dimethyl selenide induced by a resonant and non-resonant absorption (355-410 nm) and using multiphoton-ionization time-of-flight mass spectrometry, UV spectrophotometry and REMPI spectroscopy revealed that this process yields Se atoms produced from competitive dissociation channels [40]. One of these channels involved neutral CH_3_Se and CH_3 _fragments, followed by ionization of these fragments, while another channel consisted in direct ionization of (CH_3_)_2_Se with subsequent fragmentation. The rate of ionization decreases and that of fragmentation dominates at low laser intensity. The process is feasible for the formation of Se, but it is not clear whether final deposit is contaminated by hydrocarbon products (CH_3 _and C_2_H_x_) or products of their decomposition. 

KrF laser-induced photolysis of dimethyl selenide in excess of He is controlled by cleavage of both C-Se bonds, affords ethane as a very dominant (95-99 mol %) gaseous product (along with minor amounts of methane an ethene), and allows an instant formation of a white smog, which descending onto the reactor walls, creates Se films of initially white and later pink color [41]. The quantum yield of the photolysis is 0.16 Me_2_Se molecule decomposed by one absorbed photon. The photon energy at 248 nm corresponding to ca. 480 kJ Einstein^-1^ is barely sufficient to break both Se-C bonds. The preponderance of ethane among hydrocarbon products is consistent with a twofold cleavage of the C-Se bonds, which possibly occurs as a molecular extrusion of ethane (methyl fragments recombining within molecular sphere). 

**Scheme 1:**
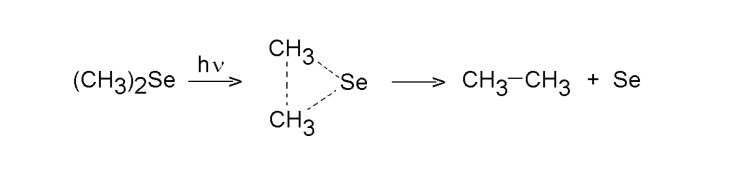
KrF laser decomposition of dimethyl selenide.

UV-Vis and XPS spectral analyses of the films reveal pure amorphous selenium and electron microscopy showed that the films have structure of isolated ca. 0.1 - 1 m-sized round-shape particles composed of 20 nm large tightly bonded dots.

The KrF laser photodissociation of dimethyl telluride [42] produces ground-state Te atoms via a single photon process. ArF laser-induced photolysis of dimethyl telluride in excess of He yields [43] methane and ethane along with a grayish fog that fills the reactor volume and descends to its bottom. The relative molar percentage of ethane (85-95 %) is in keeping with homolysis of the Te-C bond, followed by the combination of methyl radicals (as a dominant path) and with H-abstraction from (CH_3_)_2_Te by CH_3_ radicals as a very minor route. The energy delivered by the 193 nm photons corresponds to ca. 620 kJ Einstein^-1^ and is enough for cleavage of both Te-C bonds. The deposited solid is amorphous tellurium that contains some carbonaceous impurities in topmost layers.

#### 3.1.2. Diethyl selenide and diethyl telluride

**Scheme 2:**
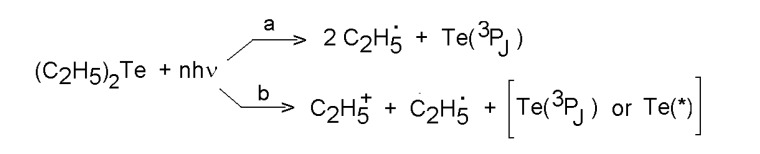
Multiple photon dissociation of diethyl telluride.

Laser photolysis of these compounds, particularly of diethyl telluride, was examined at different laser radiations. Dissociation dynamics of diethyl telluride was studied by using laser-induced fluorescence and multiple ionization spectroscopy for state-selective detection of Te atoms produced upon multiple photon dissociation of this compound. It was revealed that neutral Te atoms are mostly produced in the ground and the lowest excited states. The preferred dissociation path in the 358-395 nm excitation region was considered [44] to be formation of neutral fragments (Scheme 2, path a). Under similar excitation conditions in the 510-400 nm region, ethyl ions and neutral Te atoms were found as the main products [45] and the proposed mechanism involves dissociation of a C_2_H_5_-Te^+^ or (C_2_H_5_)_2_Te^+^ intermediate and fragments (Scheme 2, path b).

**Scheme 3:**
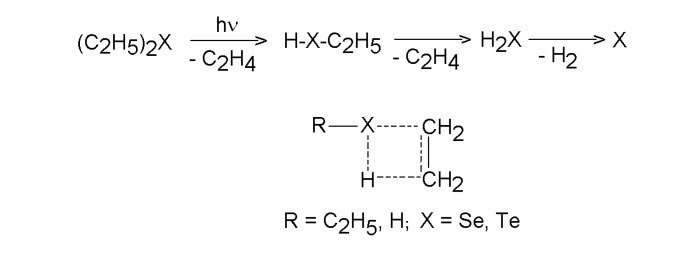
One photon dissociation of diethyl telluride and selenide.

The photodecomposition of diethyl telluride upon irradiating with ArF and KrF lasers was indicated as a one-photon process [42,46]. The KrF laser photolysis of diethyl telluride probed by a dye laser pulse tuned to specific electronic states of Te atoms indicated production of ground state Te(^3^P_2_) atoms and the result was assumed [47] to agree with the single photon dissociation into ground-state Te atoms and two ethyl radicals. However, these radical species can be rejected on the basis of analysis of hydrocarbon products. Thus the KrF and ArF laser photolysis of diethyl telluride and diethyl selenide in excess of He [48,49] proceeds as a one-photon process with initial quantum yield 0.08-0.10 (Se) and 0.4-0.5 (Te) and yields ethene (a far major product), n-butane, ethane, propane and propene (minor products). These data were accounted for by the pulse laser energy sufficient to break both C-X (X=Se, Te) bonds and by an unimportant role of cleavage of the C_2_H_5_ radical (and its subsequent combination and disproportionation reactions). In accordance with the known disproportionation (2 C_2_H_5_→ C_2_H_4_ + C_2_H_6_)/combination (2 C_2_H_5_→ C_4_H_10_) rate ratio 0.13, [50], the clean C-X homolysis would have yielded *n*-butane in a large excess over equal amounts of ethane and ethene. The observed preponderance of ethene is thus consistent with β-elimination (Scheme 3) taking place via a four-centre transition state.

The deposited Se films possess good adhesion to glass and metals, while Te films are easily removable from the surface as powder. The films do not incorporate carbon, although their topmost layers undergo oxidation on exposure to air. The morphology of Se and Te films differs: the former have a compact structure with round-shape particles of 1-4 μm size, whereas the latter are fluffy agglomerates composed of 100-200 nm-bodies. Interestingly, the Te powder has a BET specific surface around 20 m^2^ g^-1^.

#### 3.1.3. Selenophene and tellurophene

**Scheme 4:**
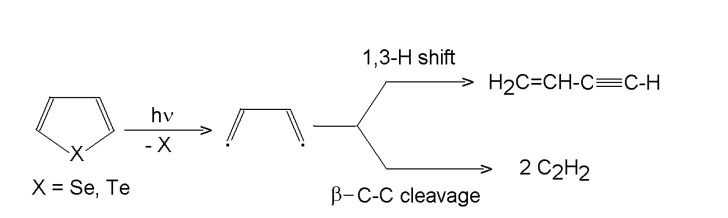
Laser photolysis of selenophene and tellurophene.

ArF and KrF laser-induced photolysis of selenophene and tellurophene (C_4_H_4_X, X = Se, Te) proceeds [51], unlike that of thiophene and furan, via cleavage of both X-C bonds and yields elemental heteroatom (Se, Te) along with 1-buten-3-yne and ethyne. The proposed mechanism involves an intermediate **^.^**CH=CH-CH=CH**^. ^**diradical that decomposes via competing 1,3-H shift to 1-buten-3-yne and β-C-C-cleavage to two molecules of ethyne (Scheme 4). The relative importance of these paths depends on the energy of the photon and on the heteroatom. Thus, the 1,3-H shift/ β-C-C-cleavage ratio are 2.3 (193 nm; M=Se), 3.6 (248 nm, M=Se), 1.4 (193 nm, M=Te) and 10.5 (248 nm, M=Te). The observed preference for the H-shift in the postulated diradical differs from the relative importance of both pathways theoretically predicted for thermally equilibrated system. It was assumed that the outstandingly greater enhancement of the 1,3-H shift when using 193 nm photons instead of 248 nm photons with tellurophene compared to that with selenophene reflects a different energy portioning between the C_4_H_4_ diradical and the two different X atoms, because it can be assumed that the greater amount of energy being carried away by the heavier Te leaves the diradical less energetic and hence less apt to the high energy β-C-C-cleavage. The leaving Te and Se atoms are inert residues, which suggests that the photolysis of selenophene and tellurophene can serve as an efficient method for the generation of the C_4_H_4_ diradical in mechanistic studies.

Both photolyses allow [52] deposition of Se and Te films which are not contaminated by carbon from hydrocarbon photolytic products. The Se films have a structure of isolated ball-shaped 2 μm particles, whereas Te films show smaller (0.3-0.5 m) particles that are agglomerated into a continuous fluffy structure. Both Se and Te films are amorphous and the Te films possess UV-Vis absorption features differring them from the known thin granular or thick Te layers.

### 3.2. IR laser thermolysis

#### 3.2.1. Dimethyl selenide and dimethyl telluride

**Scheme 5:**
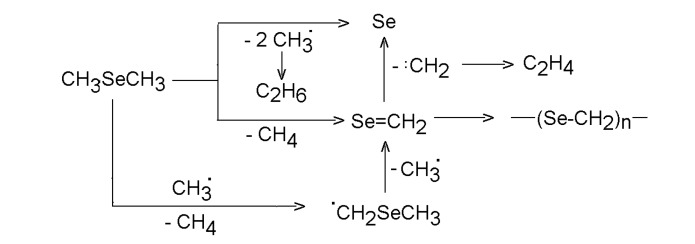
Homogeneous decomposition of dimethyl selenide.

The continuous-wave and pulsed CO_2_ laser-induced and SF_6_-photosensitized decomposition of dimethyl selenide [53] yields methane, ethene and ethane as three major products and allows deposition of a composite of elemental Se and a polymer assigned as poly(selenoformaldehyde). The formation of only three hydrocarbons, accounted for by reactions in Scheme 5, is in contrast with the observation of a number of hydrocarbons in conventional (heterogeneous) pyrolysis [35]. The occurrence of methyl radicals was proved by scavenging them with D_2_.

The solid films deposited in both processes are orange in color and differ from the pink Se films obtained upon UV laser photolysis of dimethyl selenide [41]. The films consist of ca. 1μm sized round-shape particles, ca. 2-4 μm sized oval particles and irregular 1-3 μm sized agglomerates. XPS analysis revealed that Se is present in two different chemical states which are in accord with elemental selenium and a polymer composed of Se and C atoms. The UV spectrum having a maximum at 450 nm was assigned to poly(selenoformaldehyde).

**Scheme 6:**
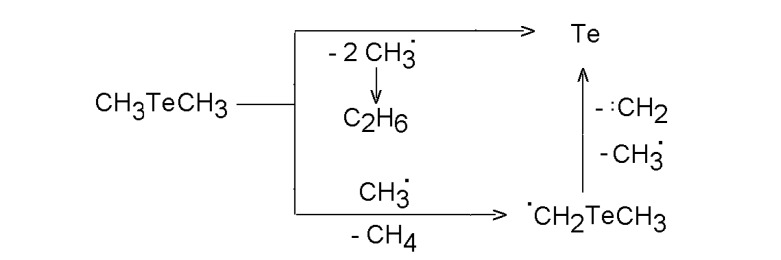
Homogeneous decomposition of dimethyl telluride.

Pulsed CO_2_ laser-induced and SF_6_ photosensitized decomposition of dimethyl telluride [54] yields ethane, methane, ethene, propane and allows deposition of nanostructured crystalline Te films. The predominance of ethane (ca 70 %) is in a great contrast with the reported dominance of methane (ca 90 %) in the conventional pyrolysis. The hydrocarbon distribution (other products: methane ~20%, ethene ~10 %) was explained by the steps in Scheme 6.

An alternative path leading to an elusive Te=CH_2 _species (polymerizing to a polymer) was ruled out by proving that the deposited solid film is elemental Te. This deposit is a crystalline ultrafine powder and consists of particles smaller than 1μm formed by 20-200 nm-sized bodies. The crystalline Te does not undergo oxidation on air, which contradicts to easy oxidation of a-Te produced by laser photolysis.

#### 3.2.2. Selenophene and tellurophene

**Scheme 7:**
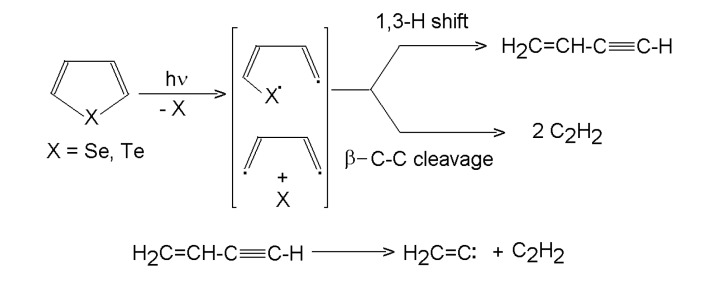
Homogeneous decomposition of tellurophene and selenophene.

The pulsed and continuous-wave CO_2_ laser induced homogeneous decomposition of selenophene and tellurophene in the presence of SF_6_ sensitizer [55] is controlled by cleavage of both X-C (X=Se, Te) bonds and yields elemental chalcogen along with 1-buten-3-yne, ethyne, ethene and buta-1,3-diene (major products, selenophene) and 1-buten-3-yne (major product, tellurophene). The higher SF_6_ content in the irradiated selenophene-SF_6_ system increases the mean effective temperature and leads to higher relative amounts of ethyne and lower amounts of 1-buten-3-yne and ethene. Scavenging experiments with D_2 _and the identification of d-isotopomers of hydrocarbon products confirmed that the extent of radical reactions in the decomposition of tellurophene is remarkably lower than in the decomposition of selenophene. Radical reactions are of low importance even in the selenophene decomposition, as 1-buten-3-yne decomposes mostly via molecular elimination of ethyne. The main reaction steps deduced on the basis of relative amounts of hydrocarbons are given Scheme 7. 

The relative importance of 1,3-H shift and β-cleavage in the C_4_H_4_ (and/or MC_4_H_4_) diradical, reflected by the 1-buten-3-yne/(0.5 ethyne) ratio depends on the SF_6_ content and is ca. 2-7 (selenophene) and 8-33 (tellurophene). It was envisaged that the greater amount of energy being carried out by the heavier Te atoms makes the C_4_H_4_ diradical produced from tellurophene more apt to follow a lower-energy (1-3 H shift) path. The orange films deposited from selenophene consisted of selenium and traces of carbon, whereas dark films deposited from tellurophene were proved to be pure Te. 

## 4. Deposition of chalcogen/polymer composite

**Scheme 8:**
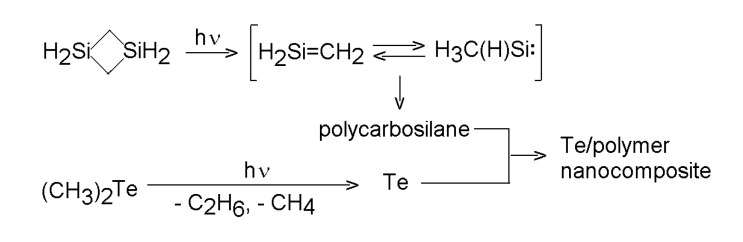
Co-photolysis of dimethyl telluride and 1,3-disilacyclobutane.

UV laser-irradiation of gaseous mixtures of Te or Se precursor together with a polymer precursor can result in the co-photolysis of both compounds and co-deposition of chalcogen and polymer. Such procedure was demonstrated for ArF laser-irradiation of a gaseous mixture of dimethyl telluride and 1,3-disilacyclobutane. The concurrent photolysis of both compounds results [42] in: (i) the expulsion of Te from dimethyl telluride and (ii) formation of a short-lived and fast polymerizing silene from 1,3-disilacyclobutane (the occurrence of the intermediate silene and its feasible gas-phase polymerization has been reported [56,57]). These reactions allow chemical vapor co-deposition of a nano-sized Te-polycarbosilane composite that contains amorphous nano-structures of tellurium stabilized against oxidation by organosilicon polymer (Scheme 8).

This protection of Te films is very important for application purposes. Very attractive tellurium films are known to decrease their reliability when exposed to atmosphere due to their oxidation and reaction with water vapor. The laser-deposited polymer-stabilized Te films contain amorphous Te protected against oxidation in their deeper layers, which represents more than 99 per cent of the bulk and the co-photolysis method can serve as an alternative to the only previously reported technique for production of polymer-stabilized Te nanoparticles using plasma enhanced chemical vapor co-deposition from diorganyl telluride and some common monomers [58].

## 5. Deposition of metal chalcogenides

Laser photolysis or laser thermolysis of a pair of gaseous chalcogen precursor and metal precursor allows simultaneous gas-phase formation of atoms (clusters) of chalcogen and metal, mutual gas-phase reaction of these species and gas-phase deposition of metal chalcogen. Simultaneous photolytic decomposition of chalcogen and metal precursors has been often used for photochemically enhanced epitaxial growth of II-VI compound semiconductors [59]. In these processes, Te precursor was decomposed together with metal (Hg, Cd) precursor in the gas phase using a laser beam parallel to hot surface and the resulting materials were produced through heterogeneous hot-surface reaction.

Purely gas-phase formation of metal chalcogenides eliminating surface assistance was achieved through homogeneous CO_2_ laser-induced, SF_6_-sensitized co-decomposition of chalcogen and metal precursor in the gas phase, mutual gas phase reaction of the formed species and deposition of the metal chalcogenide onto cold surfaces. The first example of such gas-phase synthesis of inorganic compound [60] refers to IR laser co-pyrolysis of dimethyl telluride and tetramethyltin, which results in the formation of hydrocarbons (ethane, methane, ethene) and concomitant deposition of black non-adherent solid films that were identified as containing crystalline and homogeneously distributed nanosized structures of Sn, Te and SnTe and some carbon impurities. Similarly, gas-phase synthesis of nanostructured germanium telluride has been achieved by using CO_2_ laser irradiation of gaseous (CH_3_)_4_Ge - (CH_3_)_2_Te - SF_6_ mixtures [61]. The homogeneous decomposition of both Te and Ge precursors allowed gas-phase formation and deposition of nanoscopic amorphous GeTe_2_ and crystalline GeTe.

**Scheme 9:**
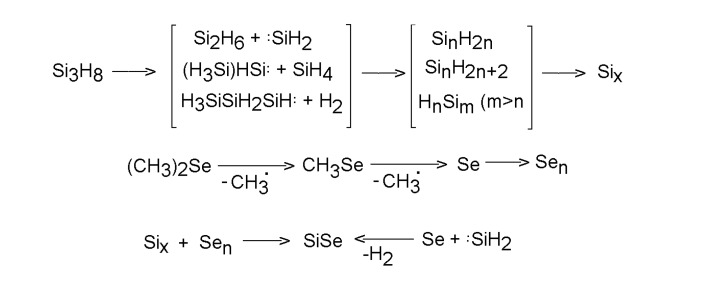
Homogeneous co-decomposition of trisilane and dimethyl selenide.

Laser-induced fluorescence (LIF)-diagnosed CO_2_ laser-irradiation of dimethyl selenide and trisilane leading to infrared multiple photon decomposition of both compounds revealed [62] gas-phase formation of SiSe. Apart from this transient, other short-lived species as silylene, methylene, Si_2_, Se_2_ and H_2_ species have been detected in the gas phase, together with final volatile hydrocarbons and Se-containing compounds (CH_3_SeH and H_2_Se) and solid Si/Se/C/H/O deposit. All these species prove the interference of dimethyl selenide and trisilane decompositions Scheme 9).

The absence of the gas-phase produced SiSe in the solid phase was assumed as caused by its polymerization and/or fast reactions with number of gas-phase produced compounds. The deposited solid liberated H_2_Se and CH_3_SeH upon exposure to air. This is in line with the presence of the Si-Se-Si and CH_3_-Si-Se- moieties contained in poly(silacarboselenides) and with their hydrolysis upon contact with air moisture.

**Scheme 10:**
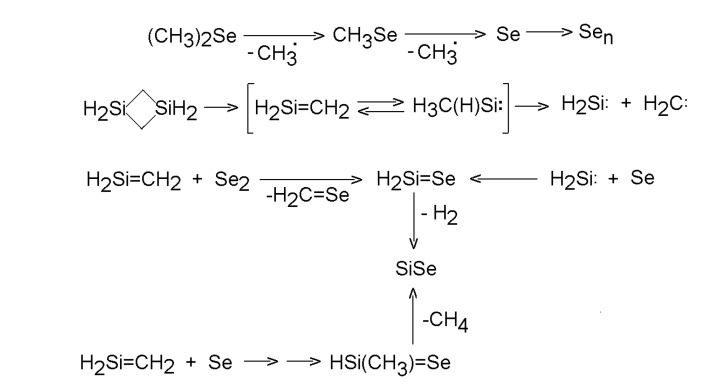
Homogeneous co-decomposition of dimethyl selenide and 1,3-disilacyclobutane.

A LIF excitation spectrum of SiSe obtained upon CO_2_ laser irradiation of gaseous mixture of 1,3-disilacyclobutane and dimethyl selenide is consistent with infrared multiple photon decomposition of both compounds and interference of both decompositions resulting in the transient formation of SiSe [63,64]. The SiSe formation was explained in terms of reaction of Se atoms with RHSi: silylenes (R = CH_3_, H) and elimination of RH from silaneselones RHSiSe (Scheme 10).

A unique laser approach to “deposition” of metal chalcogenides consists in ArF laser gas-phase photolysis of diethyl selenide and allowing Se deposit to metal surfaces at room temperature [65]. Thin coatings of Se take part in a solid-state chemical reaction with Ag, Cd, Cu, Mg and Zn surfaces to yield metal selenides. These results reveal the feasibility of room-temperature selenization of some metals and demonstrated that high temperatures previously required for bulk reaction between Se and metal are not necessary for formation of some metal selenides from thin Se films.

## 6. Summary

The laser-induced gas-phase photolytic and thermolytic decomposition of organic selenide or telluride proceeds without detrimental surface-assisted wall reactions and allows homogeneous decomposition of these compounds resulting in the gas-phase formation of volatile non-obtrusive hydrocarbons and extrusion of selenium and tellurium depositing from the gas phase as nanostructured powder or thin film. 

The laser-induced gas-phase co-photolysis and thermolysis of a mixture of organic selenide (or telluride) and polymer or IVA group element precursor allows gas-phase formation and deposition of nanostructured chalcogen/polymer composites and IVA group (Si, Ge, Sn) element chalcogenides. This is possible due to gas-phase reaction between concurrently generated chalcogen and polymer or IVA group element. This approach can find extension for gas-phase formation and deposition of more inorganic compounds and it is promising for fabrication of novel nanostructured materials.
